# Stimulus-Driven Brain Rhythms within the Alpha Band: The Attentional-Modulation Conundrum

**DOI:** 10.1523/JNEUROSCI.1633-18.2019

**Published:** 2019-04-17

**Authors:** Christian Keitel, Anne Keitel, Christopher S.Y. Benwell, Christoph Daube, Gregor Thut, Joachim Gross

**Affiliations:** ^1^Institute of Neuroscience and Psychology, University of Glasgow, Glasgow G12 8QB, UK,; ^2^Psychology, School of Social Sciences, University of Dundee, Dundee DD1 4HN, UK, and; ^3^Institut für Biomagnetismus und Biosignalanalyse, Westfälische Wilhelms-Universität, 48149 Münster, Germany

**Keywords:** alpha rhythm, entrainment, frequency tagging, phase synchronization, spatial attention, steady-state response (SSR)

## Abstract

Two largely independent research lines use rhythmic sensory stimulation to study visual processing. Despite the use of strikingly similar experimental paradigms, they differ crucially in their notion of the stimulus-driven periodic brain responses: one regards them mostly as synchronized (entrained) intrinsic brain rhythms; the other assumes they are predominantly evoked responses [classically termed steady-state responses (SSRs)] that add to the ongoing brain activity. This conceptual difference can produce contradictory predictions about, and interpretations of, experimental outcomes. The effect of spatial attention on brain rhythms in the alpha band (8–13 Hz) is one such instance: alpha-range SSRs have typically been found to increase in power when participants focus their spatial attention on laterally presented stimuli, in line with a gain control of the visual evoked response. In nearly identical experiments, retinotopic decreases in entrained alpha-band power have been reported, in line with the inhibitory function of intrinsic alpha. Here we reconcile these contradictory findings by showing that they result from a small but far-reaching difference between two common approaches to EEG spectral decomposition. In a new analysis of previously published human EEG data, recorded during bilateral rhythmic visual stimulation, we find the typical SSR gain effect when emphasizing stimulus-locked neural activity and the typical retinotopic alpha suppression when focusing on ongoing rhythms. These opposite but parallel effects suggest that spatial attention may bias the neural processing of dynamic visual stimulation via two complementary neural mechanisms.

**SIGNIFICANCE STATEMENT** Attending to a visual stimulus strengthens its representation in visual cortex and leads to a retinotopic suppression of spontaneous alpha rhythms. To further investigate this process, researchers often attempt to phase lock, or entrain, alpha through rhythmic visual stimulation under the assumption that this entrained alpha retains the characteristics of spontaneous alpha. Instead, we show that the part of the brain response that is phase locked to the visual stimulation increased with attention (as do steady-state evoked potentials), while the typical suppression was only present in non-stimulus-locked alpha activity. The opposite signs of these effects suggest that attentional modulation of dynamic visual stimulation relies on two parallel cortical mechanisms—retinotopic alpha suppression and increased temporal tracking.

## Introduction

Cortical visual processing has long been studied using rhythmic sensory stimulation (Adrian and Matthews, 1934; Walter et al., 1946; Regan, 1966). This type of stimulation drives continuous brain responses termed steady-state responses (SSRs) that reflect the temporal periodicities in the stimulation precisely. SSRs allow tracking of individual stimuli in multielement displays (Vialatte et al., 2010; Norcia et al., 2015). Further, they readily indicate cognitive biases of cortical visual processing, such as the selective allocation of attention (Morgan et al., 1996; Keitel et al., 2013; Störmer et al., 2014).

Although SSRs can be driven using a wide range of frequencies (Herrmann, 2001), stimulation at alpha band frequencies (8–13 Hz) has stirred particular interest. Alpha rhythms dominate brain activity in occipital visual cortices (Groppe et al., 2013; Keitel and Gross, 2016) and influence perception (Benwell et al., 2017, 2018; Iemi et al., 2017; Samaha et al., 2017). Researchers have therefore used alpha-rhythmic visual stimulation in attempts to align the phase of—or to entrain—intrinsic alpha rhythms and consequently provided evidence for visual alpha entrainment (Mathewson et al., 2012; Zauner et al., 2012; Spaak et al., 2014; Gulbinaite et al., 2017). These findings suggest that at least part of the SSR driven by alpha-band stimulation should be attributed to entrained alpha generators (Notbohm et al., 2016).

Some issues remain with such an account (Capilla et al., 2011; Keitel et al., 2014). For instance, experiments have consistently reported that SSR power increases when probing the effects of spatial selective attention on SSRs driven by lateralized hemifield stimuli (Müller et al., 1998a), also when using alpha-band frequencies (Kim et al., 2007; Kashiwase et al., 2012; Keitel et al., 2013). However, recent studies that used similar paradigms, but treated alpha-frequency SSRs as phase-entrained alpha rhythms, in line with an earlier study using rhythmic transcranial magnetic stimulation (Herring et al., 2015), reported the opposite effect (Kizuk and Mathewson, 2017; Gulbinaite et al., 2019). Oscillatory brain activity showed attentional modulations characteristic of the intrinsic alpha rhythm during stimulation: alpha power decreased over the hemisphere contralateral to the attended position, an effect known to be part of a retinotopic alpha power lateralization during selective spatial attention (Worden et al., 2000; Kelly et al., 2006; Thut et al., 2006; Rihs et al., 2007; Capilla et al., 2014). Briefly put, studies analyzing SSRs show a power increase, whereas studies analyzing “entrained alpha” show a power decrease with attention.

Both neural responses originate from visual cortices contralateral to the hemifield position of the driving stimuli (Keitel et al., 2013; Spaak et al., 2014). Assuming a single underlying neural process, opposite attention effects therefore seemingly contradict each other. However, results in support of alpha entrainment differed in how exactly responses to the periodic stimulation were quantified. Effects consistent with SSR modulation resulted from spectral decompositions performed on trial-averaged EEG waveforms. This approach tunes the resulting power estimate to the part of the neural response that is sufficiently time locked to the stimulation (Tallon-Baudry et al., 1996; Delorme and Makeig, 2004). Effects consistent with alpha entrainment instead typically result from averages of single-trial spectral transforms, thus emphasizing intrinsic non-phase-locked activity (Tallon-Baudry et al., 1998; Herrmann et al., 2004). Both approaches have been applied before to compare stimulus-evoked and induced brain rhythms in alpha (Moratti et al., 2007) and gamma (Tallon-Baudry et al., 1998; Picton et al., 2003; ∼40 Hz) frequency ranges. Here we focused on contrasting the attentional modulation of alpha during- and SSRs driven by an alpha-rhythmic stimulation.

We therefore compared the outcome of both approaches in a new analysis of previously reported EEG data (MATLAB analysis script and data available in Fieldtrip format on the Open Science Framework, https://osf.io/apsyf/; Keitel et al., 2017b). Participants viewed two lateralized stimuli, both flickering at alpha band frequencies (10 and 12 Hz). They were cued to focus on one of the two and perform a target detection task at the attended position. We quantified spectral power estimates according to both approaches described above from the same EEG data. Should the outcome depend on the approach taken, we expected to find the typical alpha power lateralization (contralateral < ipsilateral) when averaging single-trial power spectra. In power spectra of trial-averaged EEG, instead we expected the typical SSR power gain modulation in the opposite direction (contralateral > ipsilateral). Crucially, such an outcome would warrant a re-evaluation of stimulus-driven brain rhythms in the alpha range and intrinsic alpha as a unitary phenomenon (alpha entrainment).

## Materials and Methods

### 

#### 

##### Participants.

For the present report, we reanalyzed the EEG data of 17 volunteers recorded in an earlier study (Keitel et al., 2017a). Participants (13 women; median age, 22 years; age range, 19–32 years) declared normal or corrected-to-normal vision and no history of neurological diseases or injury. All procedures were approved by the ethics committee of the College of Science & Engineering at the University of Glasgow (application no. 300140020) and adhered to the guidelines for the treatment of human subjects in the Declaration of Helsinki. Volunteers received monetary compensation of £6/h. They gave informed written consent before participating in the experiment. Note that we excluded five additional datasets on grounds reported in the original study (four showed excessive eye movements, one underperformed in the task).

##### Stimulation.

**Figure 1.**
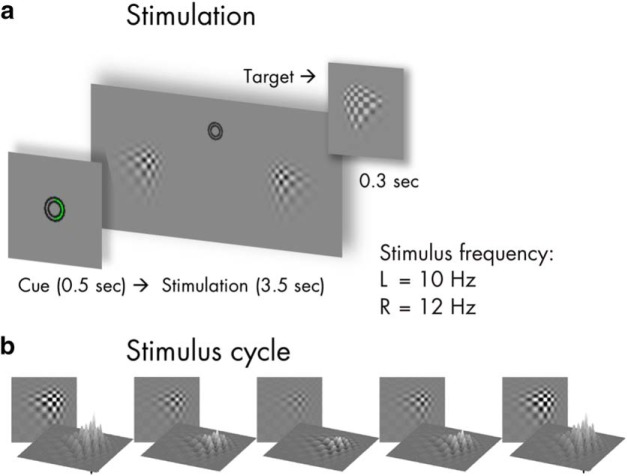
Stimulus schematics and trial time course. ***a***, shows the time course of one trial with a cue displayed for 0.5 s (here: attend right), followed by the bilateral visual stimulation for 3.5 s. Left (L) stimulus contrast fluctuated with a rate of 10 Hz and right (R) stimulus contrast at 12 Hz. Targets that participants were instructed to respond to were slightly altered versions of the stimuli (see inset) that were displayed occasionally for 0.3 s. ***b***, Rhythmic visual stimulation was achieved by a frame-by-frame adjustment of global stimulus contrast (through local luminance changes) as exemplified here in one representative cycle.

Participants viewed experimental stimuli on a computer screen (refresh rate, 100 frames/s) at a distance of 0.8 m that displayed a gray background (luminance, 6.5 cd/m^2^). Small concentric circles in the center of the screen served as a fixation point (Fig. 1). Two blurry checkerboard patches (horizontal/vertical diameter, 4° of visual angle) were positioned at an eccentricity of 4.4° from central fixation, one each in the lower left and lower right visual quadrants. Both patches changed contrast rhythmically during trials: Stimulus contrast against the background was modulated by varying patch peak luminance between 7.5 cd/m^2^ (minimum) and 29.1 cd/m^2^ (maximum).

On each screen refresh, peak luminance changed incrementally to approach temporally smooth contrast modulations as opposed to a simple on-off flicker (Andersen and Müller, 2015). Further details of the stimulation can be found in Keitel et al. (2017a). The contrast modulation followed a 10 Hz periodicity for the left stimulus and a 12 Hz periodicity for the right stimulus. Note that the experiment featured further conditions displaying quasirhythmic contrast modulations in different frequency bands. Corresponding results can be found in the original report and will not be considered in the present analysis.

##### Procedure and task.

Participants performed the experiment in an acoustically dampened and electromagnetically shielded chamber. In total, they were presented with 576 experimental trials, subdivided into eight blocks with durations of ∼5 min each. Between blocks, participants took self-paced breaks. Before the experiment, participants practiced the behavioral task (see below) for at least one block. After each block, they received feedback regarding their accuracy and response speed. The experiment was composed of eight conditions (72 trials each) resulting from a manipulation of the two factors attended position (left vs right patch) and stimulation frequency (one rhythmic and three quasirhythmic conditions) in a fully balanced design. Trials of different conditions were presented in pseudorandom order. As stated above, the present study focused on the two conditions featuring fully rhythmic stimuli. Corresponding trials (*N* = 144) were thus selected a posteriori from the full design.

Single trials began with cueing participants to attend to the left or right stimulus for 0.5 s, followed by presentation of the dynamically contrast-modulating patches for 3.5 s (Fig. 1). After patch offset, an idle period of 0.7 s allowed participants to blink before the next trial started.

To control whether participants maintained a focus of spatial attention, they were instructed to respond to occasional brief “flashes” (0.3 s) of the cued stimulus (“targets”) while ignoring similar events in the other stimulus (“distracters”). Targets and distracters occurred in one-third of all trials and up to two times in one trial with a minimum interval of 0.8 s between subsequent onsets. Detection was reported as speeded responses to flashes (recorded as the space bar presses on a standard keyboard).

##### Behavioral data recording and analyses.

Flash detections were considered a “hit” when a response occurred from 0.2 to 1 s after target onset. Delays between target onsets and responses were considered reaction times (RTs). Statistical comparisons of mean accuracies (proportion of correct responses to the total number of targets and distracters) and median RTs between experimental conditions were conducted and reported in Keitel et al. (2017a). In the present study, we did not consider the behavioral data further. Note that the original statistical analysis found that task performance in attend-left and attend-right conditions was comparable.

##### Electrophysiological data recording.

EEG was recorded from 128 scalp electrodes and digitally sampled at a rate of 512 Hz using the ActiveTwo system (BioSemi). Scalp electrodes were mounted in an elastic cap and positioned according to an extended 10–20 system (Oostenveld and Praamstra, 2001). Lateral eye movements were monitored with a bipolar outer canthus montage (horizontal electro-oculogram). Vertical eye movements and blinks were monitored with a bipolar montage of electrodes positioned below and above the right eye (vertical electro-oculogram).

##### Electrophysiological data preprocessing.

From continuous data, we extracted epochs of 5 s, starting 1 s before patch onset using the MATLAB (RRID:SCR_001622) toolbox EEGLAB (Delorme and Makeig, 2004; RRID:SCR_016333). In further preprocessing, we excluded epochs that corresponded to trials containing transient targets and distracters (24 per condition) as well as epochs with horizontal and vertical eye movements exceeding 20 μV (∼2° of visual angle) or containing blinks. For treating additional artifacts, such as single noisy electrodes, we applied the “fully automated statistical thresholding for EEG artifact rejection” (FASTER; Nolan et al., 2010). This procedure corrected or discarded epochs with residual artifacts based on statistical parameters of the data. Artifact correction used a spherical–spline-based channel interpolation. Epochs with >12 artifact-contaminated electrodes were excluded from analysis.

From 48 available epochs per condition, we discarded a median of 14 epochs for the attend-left conditions and 15 epochs for the attend-right conditions per participant with a between-subject range of 6–28 (attend-left) and 8–31 epochs (attend-right). Within-subject variation of the number of epochs per condition remained small with a median difference of three trials (maximum difference, 9 for one participant).

Subsequent analyses were performed in FieldTrip (Oostenveld et al., 2011; RRID:SCR_004849) in combination with custom-written routines. We extracted segments of 3 s starting 0.5 s after patch onset from preprocessed artifact-free epochs (5 s). Data before stimulation onset (1 s), only serving to identify eye movements shortly before and during cue presentation, were omitted. To attenuate the influence of stimulus onset-evoked activity on EEG spectral decomposition, the initial 0.5 s of stimulation were excluded. Last, because stimulation ceased after 3.5 s, we also discarded the final 0.5 s of original epochs.

##### Electrophysiological data analyses—spectral decomposition.

Artifact-free 3 s epochs were converted to scalp current densities (SCDs), a reference-free measure of brain electrical activity (Ferree, 2006; Kayser and Tenke, 2015), by means of the spherical spline method (Perrin et al., 1987) as implemented in FieldTrip (function *ft_scalpcurrentdensity*, method “spline”; lambda = 10^−4^). Detrended (i.e., mean and linear trend removed) SCD time series were then Tukey tapered and subjected to Fourier transforms while using zero padding to achieve a frequency resolution of 0.25 Hz. Crucially, from resulting complex Fourier spectra, we calculated two sets of aggregate power spectra with slightly different approaches. First, we calculated power spectra as the average of squared absolute values of complex Fourier spectra (*Z*) as follows:

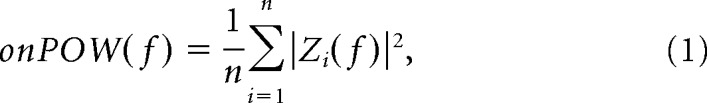
 where onPOW is the classical power estimate for ongoing (intrinsic) oscillatory activity for frequency *f*, and *n* is the number of trials. Second, we additionally calculated the squared absolute value of the averaged complex Fourier spectra according to the following:

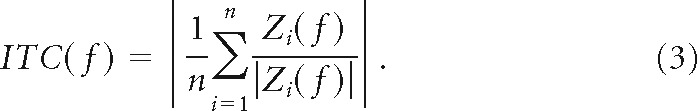
 The formula yields evoPOW, or evoked power, an estimate that is identical with the frequency tagging standard approach of averaging per-trial EEG time series before spectral decomposition. This step is usually performed to retain only the truly phase-locked response to the stimulus (Tallon-Baudry et al., 1996). Note that both formulas differ only in the order in which weighted sums and absolute values are computed. Also note that Equation 2 is highly similar to the calculation of intertrial phase coherence (ITC), a popular measure of phase locking (Cohen, 2014; Gross, 2014; van Diepen and Mazaheri, 2018). ITC calculation additionally includes a trial-by-trial amplitude normalization. To complement our analysis, we thus quantified ITC according to the following equation:

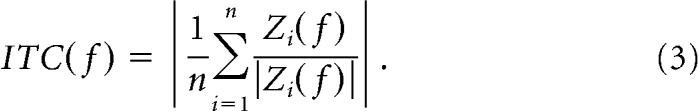
 For further analyses, power spectra were normalized by converting them to decibel scale (i.e., taking the decadic logarithm), then multiplying by 10 (hereafter termed “log power spectra”). ITC was converted to ITCz to reduce the bias introduced by differences in trial numbers between conditions (Bonnefond and Jensen, 2012; Samaha et al., 2015).

##### Alpha power—attentional modulation and lateralization.

**Figure 2.**
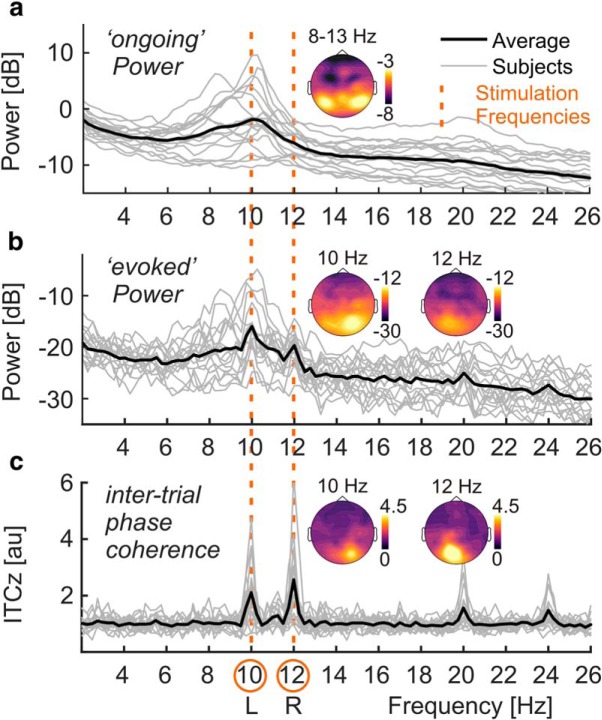
EEG spectral decomposition. ***a***, Power spectra collapsed across conditions and all electrode positions below the sagittal midline for single subjects (light gray lines) and group averages (strong black line). Note the characteristic alpha peaks in the frequency range of 8–13 Hz. Inset, Scalp map shows topographical distribution of alpha power on a dB scale based on scalp current densities. ***b***, Same as in ***a*** but for evoked power. Distinct peaks are visible at stimulation frequencies of 10 and 12 Hz (dashed vertical orange lines across plots). Inset, Scalp maps show topographical distributions of SSR power at 10 and 12 Hz on a dB scale. Note the difference in scale between ongoing power (***a***) and evoked power (***b***). ***c***, Same as in ***a*** but for inter-trial phase-locking (ITCz). Inset, Scalp maps show topographical distributions of SSR ITCz at 10 and 12 Hz.

**Figure 3.**
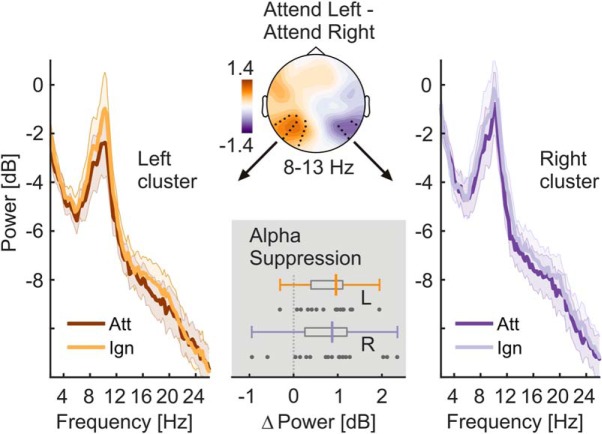
Allocation of spatial attention produces retinotopic alpha power modulation. The scalp map (top, center) depicts alpha power lateralization (attend left − attend right conditions) on a dB scale. Black dots indicate left- and right-hemispheric electrode clusters that showed a consistent difference in group statistics (two-tailed cluster-based permutation tests). Left and right spectra illustrate alpha-power differences in respective clusters when the contralateral hemifield was attended (Att) versus ignored (Ign). Shaded areas indicate standard errors of the mean. The bottom gray inset depicts the distribution of individual alpha power suppression effects (ignored − attended) within left (L) and right (R) hemisphere clusters in the 8–13 Hz band. Boxplots indicate interquartile ranges (boxes) and medians (colored vertical intersectors). Dots below show individual effects (1 dot = 1 participant).

Spectra of onPOW, pooled over both experimental conditions and all electrodes, showed a prominent peak in the alpha frequency range (Fig. 2). We used the mean log ongoing power across the range of 8–13 Hz to assess intrinsic alpha power modulations by attention. Analyzing attend-right and attend-left conditions separately yielded two alpha power topographies for each participant. These were compared by means of cluster-based permutation statistics (Maris and Oostenveld, 2007) using *N* = 5000 random permutations. We clustered data across channel neighborhoods with an average size of 7.9 channels that were determined by triangulated sensor proximity (function *ft_prepare_neighbours*, method “triangulation”). The resulting probabilities (*p* values) were corrected for two-sided testing. Subtracting left-lateralized (attend-left conditions) from right-lateralized (attend-right) alpha power topographies, we found a right-hemispheric positive and a left-hemispheric negative cluster of electrodes that was due to the retinotopic effects of spatial attention on alpha power lateralization (Fig. 3), similar to an earlier reanalysis of the other conditions of this experiment (Keitel et al., 2018).

Finally, we tested the difference between attend-left and attend-right conditions (i.e., attention effects for left- and right-hemispheric clusters separately). To this end, we submitted alpha power differences (contralateral hemifield attended − ignored) to Bayesian one-sample *t* tests against zero (Rouder et al., 2009). Attention effects were further compared against each other by means of a Bayesian paired-samples *t* test as implemented in JASP (JASP-Team, 2018; RRID:SCR_015823) with a Cauchy prior scaled to *r* = 0.5, putting more emphasis on smaller effects (Rouder et al., 2012; Schönbrodt and Wagenmakers, 2018).

This procedure allowed us to quantify the evidence in favor of the null versus the alternative hypothesis (H_0_ vs H_1_). For each test, the corresponding Bayes factor (called BF_10_) showed evidence for H_1_ (compared with H_0_) if it exceeded a value of 3, and no evidence for H_1_ at BF_10_ < 1, with the intervening range of 1–3 termed “anecdotal evidence” by convention (Wagenmakers et al., 2011). Inversing BF_10_, to yield a quantity termed BF_01_, served to quantify evidence in favor of H_0_ on the same scale. For BF_10_ and BF_01_, values <1 were taken as inconclusive evidence for either hypothesis. Note that, for the sake of brevity, we report errors in BF estimates only when exceeding 0.001%.

##### SSR power—attentional modulation.

Spectra of evoked power, pooled over both experimental conditions and all electrodes, revealed periodic responses to the two stimuli at the respective stimulation frequencies, 10 and 12 Hz (Fig. 2). Therefore, we assessed attention effects for these two spectral SSR representations. Two separate cluster-based permutation tests, one for each stimulation frequency, contrasted evoked power topographies between attended and ignored (other stimulus attended) conditions. Two-sided tests were performed with the same parameters as for alpha power (see above).

Again, we found one electrode cluster carrying systematic attention effects per frequency. As for alpha, SSR power from these two clusters were subjected to separate Bayesian one-sample *t* tests against zero (one-sided, attended > ignored) and compared against each other by means of a Bayesian paired-sample *t* test (two-sided).

##### SSR intertrial phase coherence—attentional modulation.

We also evaluated a pure measure of neural phase locking to the stimulation, SSR ITC, because evoked power can be regarded as a hybrid measure depending on both the amplitude of the underlying rhythmic response and the consistency of its phase across trials. ITC indicates only the latter as SSRs are set to unit amplitude before summing across trials (see Eq. 3). ITC spectra, pooled over both experimental conditions and all electrodes, showed distinct neural phase locking at the respective driving frequencies, 10 and 12 Hz (Fig. 2). Cluster-based permutation testing confirmed topographic regions that showed systemic gain effects in ITC. Subsequently, the same Bayesian inference was applied to data from these clusters as for SSR power.

##### Correlation of alpha and SSR attention effects—group level.

As a consequence of our counterintuitive finding that SSR attention effects appeared strongest over occipital regions ipsilateral to the driving stimulus (see SSR power and intertrial phase locking—attentional modulation below, in Results), we explored a posteriori whether these effects could be explained by ipsilateral increases in alpha power during focused attention. We correlated attention effects on alpha and SSR power using Bayesian inference (rank correlation coefficient Kendall's τ*_b_*; β prior = 0.75) to test for a positive linear relationship. More specifically, we correlated the left-hemispheric alpha power suppression (ignored − attended) with the 10 Hz SSR (evoked) power attention effect (attended − ignored) and the right-hemispheric alpha power suppression with the 12 Hz SSR power attention effect. We opted for these combinations because the corresponding effects overlapped topographically (see Results). Along with the correlation coefficient ρ, we report its 95% credible interval (95% CrI).

We also probed the linear relationship between alpha power and SSR ITC attention effects. Because ITC gains were not clearly lateralized, we collapsed gain effects (attended − ignored) across both stimulation frequencies and correlated these with a hemisphere-collapsed alpha suppression index. This index was quantified as the halved sum of left- and right-hemispheric suppression effects as retrieved from significant clusters in the topographical analysis of alpha power differences (attend left − attend right), shown in Figure 3. Again, we expected a positive correlation here if alpha power suppression influenced phase locking to visual stimulation. For means of comparison, we repeated this analysis with attention effects on SSR power collapsed across frequencies.

##### Alpha and SSR attention effects—subject level regression.

The relationship between alpha power (lateralization) and SSR attentional modulation was further subjected to a more fine-grained analysis considering within-subject variability across single trials and allowing for a better control of between-subject differences in alpha and SSR power. We assumed that if the SSR attention effect (i.e., the ipsilateral SSR power gain) was a mere consequence of the colocalized alpha power increase, then these two effects should covary across trials. For this analysis, we recalculated single-trial alpha power and SSR-evoked power/ITC estimates at each EEG sensor and for both conditions in each subject based on the same artifact-removed EEG epochs and using the same spectral decomposition as described above. Because ITC is not defined for single trials, we used a jackknife approach that computed single-trial estimates in a leave-one-out procedure and allowed for the subsequent evaluation of intertrial variability (Richter et al., 2015). For consistency, we computed similar alpha-power jackknife estimates. From these estimates, we calculated attention effects as all possible pairwise differences between trials of different conditions (attend left vs attend right), yielding distributions of alpha power hemispheric lateralization and SSR-evoked power/ITC attentional modulation (for 10 and 12 Hz SSRs separately). To validate this approach, we used it to reproduce the alpha power and SSR attention effects described below (data not shown, reproducible via code in on-line repository; Keitel et al., 2017b).

We then tested for a linear relationship between both *z*-scored measures by subjecting them to a robust linear regression (MATLAB function “robustfit,” default options), performed for each EEG sensor separately. The obtained subject-specific regression coefficients β (slopes) were entered into a group statistical test. We tested slopes against zero (i.e., no linear relationship) by means of cluster-based permutation tests (two-tailed), clustering across EEG sensors. Four tests were performed in total; one for each regression of alpha power lateralization with SSR-evoked power or SSR ITC attentional modulation, and separately for 10 and 12 Hz SSR, respectively. This procedure was supplemented by sensor-by-sensor Bayesian *t* tests (Rouder et al., 2009; Schwarzkopf, 2015) to quantify the evidence in favor of a linear versus no relationship (see Materials and Methods, Alpha power—attentional modulation and lateralization regarding Bayesian inference).

## Results

### Ongoing alpha power—attentional modulation and lateralization

The power of the ongoing alpha rhythm lateralized with the allocation of spatial attention to left and right stimuli. A topographic map of the differences in alpha power between attend-left and attend-right conditions shows significant left- and right-hemispheric electrode clusters (Fig. 3). These clusters signify retinotopic alpha-power modulation when participants attended to left versus right stimulus positions (right cluster: *t*_sum_ = −21.454, *p* = 0.026; left cluster: *t*_sum_ = 81.264, *p* = 0.002). The differences are further illustrated in power spectra pooled over electrodes of each cluster (Fig. 3). As predicted, the alpha power at each cluster was lower when participants attended to the contralateral stimulus. Bayesian inference confirmed the alpha-power attention effect for the right (mean = 0.806 dB; SEM = 0.216; BF_10_ = 21.17) and left cluster (mean = 0.790 dB; SEM = 0.133; BF_10_ = 906.36). Both effects were of comparable magnitude (BF_01_ = 4.009 ± 0.007).

### SSR power and intertrial phase locking—attentional modulation

**Figure 4.**
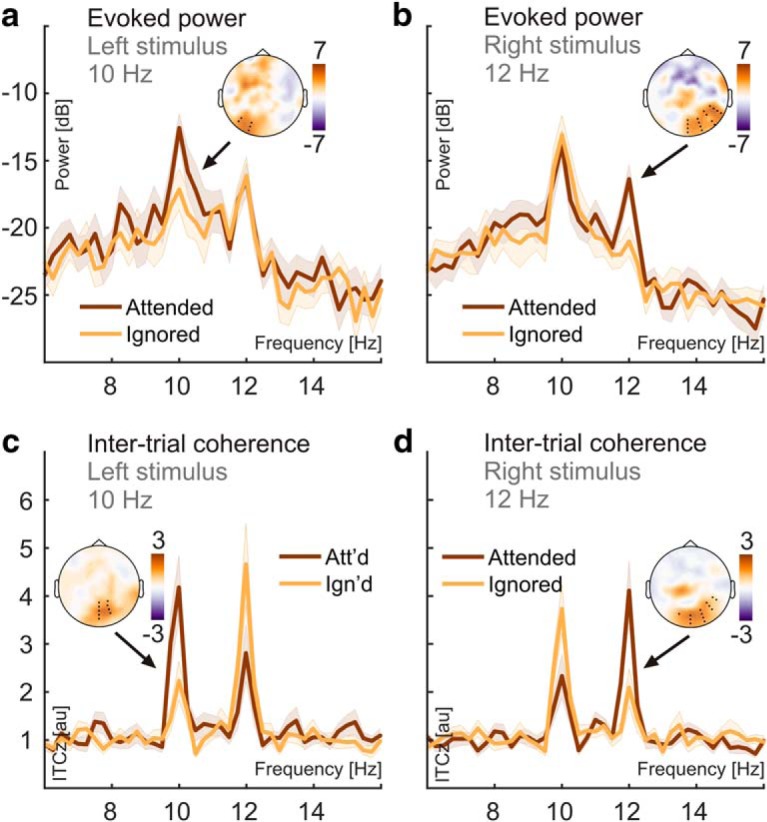
Attention effects on SSR evoked power and SSR intertrial phase coherence. ***a***, SSR evoked power spectra show systematic power differences at the presentation frequency (10 Hz) of the left stimulus when it was attended (dark red) versus ignored (orange). Shaded areas indicate standard errors of the mean. The inset scalp map illustrates the topographical distribution of the attention effects. Power spectra were averaged across electrodes (black dots in scalp maps) that showed consistent attention effects in group statistics (two-tailed cluster-based permutation tests) for attended and ignored conditions separately. ***b***, Same as in ***a*** but for the 12 Hz stimulus presented in the right visual hemifield. ***c***, ***d***, Same as in ***a*** and ***b*** but using ITCz as a measure of SSR intertrial phase coherence.

Crucially, we found the opposite pattern when looking at SSRs (i.e., the exact same data but with a slightly different focus on oscillatory brain activity that was time locked to the stimulation; compare formulas 1 and 2): SSRs showed increased power when the respective driving stimulus was attended versus ignored (Fig. 4). The power of neural responses evoked by our stimuli (SSRs) was at least one order of magnitude smaller than ongoing alpha power on average (difference > 10 dB; i.e., between 10 and 100 times). Nevertheless, SSRs could be clearly identified as distinct peaks in (evoked) power and ITC spectra. Consistent with the retinotopic projection to early visual cortices, topographical distributions of both measures showed a focal maxima contralateral to the respective attended stimulus positions (Fig. 2). Counterintuitively though, maximum attention effects on SSR power did not coincide topographically with sites that showed maximum SSR power overall (compare scalp maps Figs. 2, 4). Also, due to their rather ipsilateral scalp distributions (with respect to the attended location), SSR attention effects did not match the topographies of attention-related decreases in ongoing alpha power (compare scalp maps, Figs. 3, 4). The 10 Hz SSR driven by the left-hemifield stimulus showed a left-hemispheric power increase when attended (*t*_sum_ = 15.837, *p* = 0.059). Similarly, attention increased the power of the 12 Hz SSR driven by the right-hemifield stimulus in a right-hemispheric cluster (*t*_sum_ = 53.282, *p* < 0.001). Bayesian inference confirmed the attention effect on 10 Hz (mean = 3.727 dB; SEM = 0.919; BF_10_ = 37.05) and 12 Hz SSR power (mean = 4.473 dB; SEM = 0.841; BF_10_ = 329.75) averaged within clusters. Both effects were of comparable magnitude (BF_01_ = 3.443 ± 0.005).

SSR phase-locking (quantified as ITCz) also increased with attention to the respective stimulus. In contrast to evoked power, topographical representations of these effects showed greater overlap with the sites that showed maximum phase locking in general (Fig. 4). For both frequencies, ITCz increased in central occipital clusters (10 Hz: *t*_sum_ = 41.351, *p* = 0.004; 12 Hz: *t*_sum_ = 31.116, *p* = 0.012). Again, Bayesian inference confirmed the attention effect on 10 Hz (mean = 1.386 a.u.; SEM = 0.297; BF_10_ = 105.71, one-sided) and 12 Hz ITCz (mean = 1.824 a.u.; SEM = 0.451; BF_10_ = 36.11, one-sided). Evidence for a greater attention effect on 12 Hz than on 10 Hz ITC remained inconclusive (BF_10_ = 0.473).

### Correlation of alpha and SSR attention effects—group level

**Figure 5.**
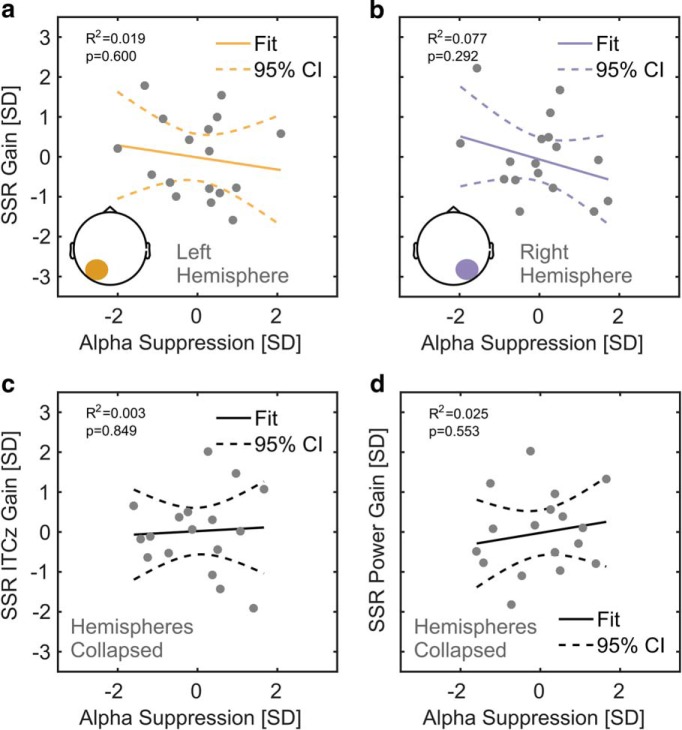
Relationships between attention effects on alpha power and SSRs. ***a***, Individual 10 Hz (left stimulus) SSR evoked power gain (attended − ignored; *z*-scored, *y*-axis) as a function of alpha suppression (ignored − attended; *z*-scored, *x*-axis) in overlapping left-hemispheric parieto-occipital electrode clusters. Gray dots represent participants. Colored lines depict a straight line fit and its confidence interval (dashed lines). The goodness of fit of the linear model provided as *R*^2^ along with corresponding *p* value. As confirmed by additional tests, both attention effects do not show a positive linear relationship that would be expected if the ipsilateral SSR power gain effect was a consequence of the ipsilateral alpha suppression. ***b***, Same as in ***a***, but for the 12 Hz SSR driven by the right stimulus in overlapping right-hemispheric parieto-occipital electrode clusters. ***c***, ***d***, Similar to ***a*** but for attention-related gain effects on SSR ITCz (*z*-scored, *y*-axis) in ***c*** and gain effects on SSR evoked power in ***d***, both collapsed across electrode clusters showing 10 and 12 Hz SSR attention effects. Alpha suppression was collapsed across left- and right-hemispheric electrode clusters (Fig. 3).

Last, we tested whether the SSR attention gain effects were mere reflections of the topographically coinciding ipsilateral ongoing alpha power increase during focused attention that co-occurred with the contralateral ongoing alpha-power decrease (Fig. 3). Speaking against this account, Bayesian inference provided moderate evidence against the expected positive correlations between the left-hemispheric alpha attention effect and the 10 Hz SSR attention effect (τ*_b_* = −0.221, 95% CrI = [0.002, 0.269]; BF_01_ = 5.811) and between the right-hemispheric alpha attention effect and the 12 Hz SSR attention effect (τ*_b_* = −0.088, 95% CrI = [0.004, 0.315]; BF_01_ = 3.904). These relationships are further illustrated by corresponding linear fits in Figure 5.

Following this analysis, we further explored the relationship between spatially nonoverlapping decreases in alpha-power contralateral to the attended position and the ipsilateral SSR power gain effects. For the lack of a specific hypothesis about the sign of the correlation in this case, we quantified the evidence for any relationship (two-sided test). The results remained inconclusive for a correlation between the left-hemispheric alpha attention effect and the right-hemispheric 12 Hz SSR attention effect (τ*_b_* = 0.235; 95% CrI = −0.110 to 0.487; BF_01_ = 1.280) and between the right-hemispheric alpha attention effect and the left-hemispheric 10 Hz SSR attention effect (τ*_b_* = 0.103l; 95% CrI = −0.218 to 0.383; BF_01_ = 2.400).

Finally, we repeated this analysis for attention effects on ITC. Because SSR ITC attention effects did not show a clear topographical lateralization (Fig. 4), they were collapsed across driving frequencies (10 and 12 Hz). Again, findings were inconclusive when looking into the correlation between these aggregate SSR ITC gain effects and a hemisphere-collapsed alpha suppression index (τ*_b_* = −0.059; 95% CrI = −0.349 to 0.251; BF_01_ = 2.653). Correlating collapsed attention effects of SSR-evoked power with the same pooled alpha suppression index yielded identical results regarding the rank correlation (see also Fig. 5, linear fits).

### Alpha and SSR attention effects–subject level regression

**Figure 6.**
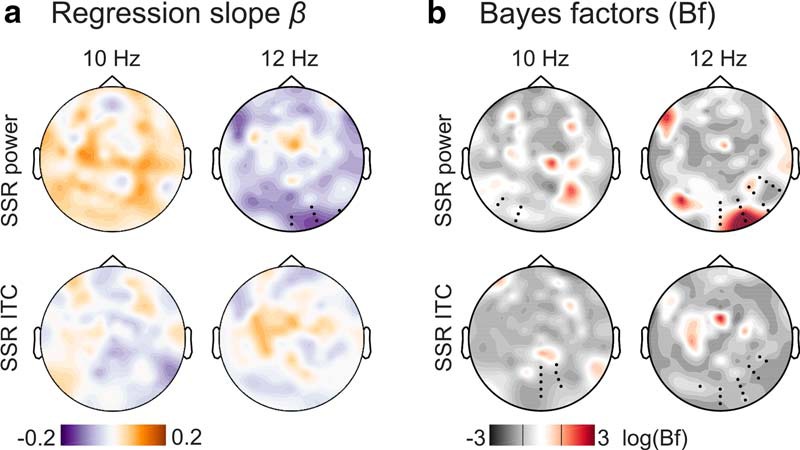
Summary of subject-level analysis of the linear relationship between alpha power and SSR attentional modulation. ***a***, The topographical distribution of group-averaged (*N* = 17) regression coefficients β (slopes) for SSR evoked power (top row) and SSR intertrial coherence (ITC, bottom row), separated by SSR frequencies of 10 Hz (left column) and 12 Hz (right column). Hot colors indicate a positive linear relationship, and cool colors a negative relationship. Black dots in the top right panel indicate a cluster of electrodes showing a systematic effect (*p* < 0.05, cluster-based permutation test) absent in tests illustrated in the other three panels. ***b***, Results of sensor-by-sensor group-level Bayesian inference (Bayesian *t* tests) of regression slopes against zero, plotted as topographies on a log(BF_10_) scale. Plots arranged as in ***a***. Red colors indicate stronger evidence for H_1_, and gray colors indicate stronger evidence for H_0_. Black lines in the color scale below scalp maps denote thresholds that signal moderate evidence for H_0_ (log(1/3) = −1.099) or H_1_ (log(3) = −1.099) by convention. Superimposed black dots indicate clusters showing systematic attention effects on SSR evoked power/ITC as depicted in Figure 4 for comparison.

A more fine-grained analysis of single-trial covariation of alpha power lateralization and SSR gain effects during focused spatial attention largely corroborated the group-level results. Clustering across EEG sensors, we found that only the 12 Hz SSR-evoked power attention effect and alpha lateralization covaried systematically across participants at occipital sites (permutation test, *t*_sum_ = −17.517, *p* = 0.023). The negative sign of the slope, however, contradicted the expected positive relationship (Fig. 6*a*). Neither 10 Hz SSR-evoked power nor SSR ITC (both frequencies) revealed similar systematic relationships with alpha power.

Additionally, we used Bayesian inference on the distributions of individual regression slopes (indicating the linear relationship between alpha and SSR attention effects) by sensor to quantify the plausibility of either H_1_ or H_0_ in scalp maps (Fig. 6*b*). We further overlaid these scalp maps with electrode clusters showing SSR attention effects (compare Fig. 4). Average BFs within clusters indicated that evidence for or against any type of linear relationship remained inconclusive for 10 Hz (mean BF_01_ = 1.422; BF_01_ range = 0.639–2.343) and 12 Hz SSR-evoked power (mean BF_01_ = 1.245; BF_01_ range = 0.153–3.673), although it should be mentioned that the 12 Hz cluster contained a local maximum (BF_10_ = 1/BF_01_ = 6.534) that coincided topographically with the effect identified by the cluster-based permutation test. For ITC, evidence favored H_0_ i.e., the absence of any relationship with 10 Hz (mean BF_01_ = 3.040; BF_01_ range = 1.861–4.014), and 12 Hz SSR (BF_01_ = 3.030; BF_01_ range = 1.391–4.016) was three times more likely given our data.

Our findings show a fine distinction between SSR-evoked power and ITC gain effects with respect to a possible connection to alpha lateralization in that only the latter provided conclusive evidence against such a relationship. As a likely explanation, SSR-evoked power still contains residual alpha activity that confounds tests for covariation. Conversely, the single-trial power normalization step undertaken during the calculation of SSR ITC makes it less susceptible to this confound. Together, the findings of this analysis do not support a positive linear relationship of alpha lateralization and SSR gain effects (especially on ITC). Therefore, it is unlikely that the counterintuitive topography of SSR attentional modulation is a reflection of alpha power lateralization during focused spatial attention.

## Discussion

We found that two common spectral measures of alpha-band EEG during alpha-rhythmic visual stimulation reflect the effects of spatial attention with opposite signs. In the following, we discuss how this finding supports the notion of two complementary neural mechanisms governing the cortical processing of dynamic visual input.

### Analysis approach determines sign of attentional modulation

When focusing on the spectral representation of ongoing EEG power, we observed the prototypical broad peak in the alpha frequency range (8–13 Hz; Fig. 2). Moreover, alpha power decreased over the hemisphere contralateral to the attended stimulus position, indicating a functional disinhibition of cortical areas representing task-relevant regions of the visual field (Worden et al., 2000; Kelly et al., 2006; Thut et al., 2006). Concurrently, alpha power increased over the ipsilateral hemisphere, actively suppressing irrelevant and possibly distracting input (Rihs et al., 2007; Capilla et al., 2014).

A second approach focused on the SSRs (i.e., strictly stimulus-locked rhythmic EEG components). As in classical frequency-tagging studies, we found spectrally distinct SSRs at the stimulation frequencies (here 10 and 12 Hz). These two concurrent rhythmic brain responses thus precisely reflected the temporal dynamics of the visual stimulation. Notably, SSR-evoked power was between one and two orders of magnitude (10–100 times) lower than ongoing-alpha power. Smaller evoked power also explained why SSRs remained invisible in spectra of ongoing activity. They were likely masked by the broad alpha peak (Fig. 2; Covic et al., 2017). Note that this is a result of the relatively low-intensity stimulation used here. Stimulation of higher intensity can evoke SSRs that are readily visible in power spectra of ongoing activity (Gulbinaite et al., 2019).

Crucially, we examined SSRs for effects of focused spatial attention. Visual cortical regions contralateral to the respective driving stimuli showed maximum SSR-evoked power. We would expect to observe a decrease in SSR-evoked power with attention (Kizuk and Mathewson, 2017; Gulbinaite et al., 2019) under the assumption that SSRs are frequency-specific neural signatures of a local entrainment of intrinsic alpha generators (Spaak et al., 2014; Notbohm et al., 2016) and exhibit similar functional characteristics. Instead, we found that SSR-evoked power increased in line with earlier reports (Kim et al., 2007; Kashiwase et al., 2012; Keitel et al., 2013).

Note, however, that these attentional gain effects did not coincide topographically with scalp locations of maximum SSR-evoked power (Fig. 4). They were most pronounced over hemispheres ipsilateral to the position of the respective driving stimuli and thus colocalized with ipsilateral alpha power increases (Fig. 3). Two control analyses showed that these effects were unlikely to be related (Figs. 5, 6). We have described the apparent counterintuitive lateralization of this effect before (Keitel et al., 2017a) when comparing scalp distributions by means of attended − unattended contrasts (Keitel et al., 2017a). In that case, expecting attention effects to emerge at sites of maximum SSR power entails the implicit assumption that attention acts only as a local response gain mechanism. Alternatively, neural representations of attended stimuli could access higher-order visual processing (Lithari et al., 2016), and a gain in spatial extent could then produce seemingly ipsilateral effects when evaluating topographical differences, as observed here. However, previous cortical source reconstructions of SSRs in lateralized stimulus situations have unequivocally localized maximum effects of visuo - spatial attention to contralateral visual cortices (Müller et al., 1998b; Lauritzen et al., 2010; Keitel et al., 2013). Considering the limited spatial resolution of EEG, and that SSR intertrial phase coherence showed yet another nonlateralized topographical distribution for gain effects (Fig. 4), warrants a dedicated neuroimaging analysis of the underlying cortical sources that generate these attentional modulations.

### Opposite but co-occurring attention effects suggest interplay of distinct attention-related processes

Our analysis compared attention effects between “ongoing” spectral power within the alpha frequency band and a quantity termed SSR evoked power that is commonly used in frequency-tagging research (Colon et al., 2012; Porcu et al., 2013; Störmer et al., 2014; Walter et al., 2016; Martinovic and Andersen, 2018). This term is somewhat misleading because it conflates a power estimate with the consistency of the phase of the SSR across trials of the experiment. ITC has been used to quantify SSRs before (Ruhnau et al., 2016). It is closely related to evoked power but involves an extra normalization term that abolishes (or at least greatly attenuates) the power contribution (Cohen, 2014; Gross, 2014). Note that in a noisy, finite signal such as the typical seconds-long EEG epoch, there will be a positive relationship between the power and intertrial phase consistency at any frequency, as is shown by the greater than zero noise floor in our ITC spectra (Fig. 4). Also note that ITC only measures SSRs meaningfully if the neurophysiological signal contains a periodic component at the stimulation frequency.

The effects of attention on SSR-evoked power and ITC are typically interchangeable (Covic et al., 2017; Keitel et al., 2017a). In fact, increased ITC, or phase synchronization, has been considered the primary effect of attention on stimulus-driven periodic brain responses (Kim et al., 2007; Kranczioch, 2017). Looking at spectral power and ITC separately, as two distinct aspects of rhythmic brain activity, therefore resolves the attentional modulation conundrum: seemingly opposing attention-related effects likely index different but parallel influences on cortical processing of rhythmic visual input. To avoid confusion, we therefore suggest opting for ITC (or related measures; e.g., the cosine similarity index; Chou and Hsu, 2018) instead of evoked power to evaluate SSRs.

Incorporating our findings into an account that regards SSRs primarily as stimulus-driven entrainment of intrinsic alpha rhythms would require demonstrating how a decrease in alpha-band power (i.e., the contralateral alpha suppression) can co-occur with increased SSR phase synchronization. Alternatively, stimulus-locked (evoked) and intrinsic alpha rhythms could be considered distinct processes (Freunberger et al., 2009; Sauseng, 2012). Consequentially, alpha range SSRs could predominantly reflect an early cortical mechanism for the tracking of fluctuations in stimulus-specific visual input per se (Keitel et al., 2017a) without the need to assume entrainment (Capilla et al., 2011; Keitel et al., 2014).

The underlying neural mechanism might similarly work for a range of rhythmic and quasirhythmic stimuli owing to the fact that visual cortex comprises a manifold of different feature detectors that closely mirror changes along the dimensions of color, luminance, contrast, spatial frequency, and more (Buracas et al., 1998; Blaser et al., 2000; Martinovic and Andersen, 2018). Most importantly, for quasirhythmic sensory input, attention to the driving stimulus may increase neural phase locking to the stimulus to allow for enhanced tracking of its dynamics (i.e., increased fidelity). This effect has been observed for quasirhythmic low-frequency visual speech signals (Crosse et al., 2015; Park et al., 2016; Hauswald et al., 2018) and task-irrelevant visual stimuli at attended versus ignored spatial locations (Keitel et al., 2017a).

Concurrent retinotopic biasing of visual processing through alpha suppression and stronger neural phase locking to attended stimuli could therefore be regarded as complimentary mechanisms. Both could act to facilitate the processing of behaviorally relevant visual input in parallel. In this context, SSRs would constitute a special case and easy-to-quantify periodic signature of early visual cortices tracking stimulus dynamics over time. Intrinsic alpha suppression instead may gate the access of sensory information to superordinate visual-processing stages (Jensen and Mazaheri, 2010; Zumer et al., 2014) and enhanced ipsilateral alpha power may additionally attenuate irrelevant and possibly distracting stimuli at ignored locations (Capilla et al., 2014).

A neural implementation may work like this: during rest or inattention, occipital neuronal populations synchronize with a strong internal, thalamocortical pacemaker (alpha). During attentive processing of sensory input, retinotopic alpha suppression releases specific neuronal subpopulations from an internal reign and allows them to track the stimulus dynamics at attended locations. A related mechanism has been observed in the striatum, where local field potentials are dominated by synchronous oscillatory activity across large areas (Courtemanche et al., 2003). However, during task performance focal neuronal populations were found to disengage from this global synchronicity in a consistent and task-specific manner. At the level of EEG/MEG recordings, such a mechanism could lead to a task-related decrease of oscillatory power but an increase of coherence or ITC, as observed in the current study and previously in the sensorimotor system (Gross et al., 2005; Schoffelen et al., 2005, 2011).

Although such an account challenges the occurrence of strictly stimulus-driven alpha entrainment, it may still allow alpha to exert temporally precise top-down influences during predictable and behaviorally relevant rhythmic stimulation—a process that itself could be subject to entrainment (Thut et al., 2011; Nobre et al., 2012; Haegens and Zion Golumbic, 2018; Zoefel et al., 2018).

### Conclusion

Our findings reconcile seemingly contradictory findings regarding spatial attention effects on alpha-rhythmic activity, assumed to be entrained by periodic visual stimulation, and SSRs. Focusing on spectral power or phase consistency of the EEG during visual stimulation yielded reversed attention effects in the same dataset. Our findings encourage a careful and consistent choice of measures of ongoing brain dynamics (here power) or measures of stimulus-related activity (here ITC), that should be critically informed by the experimental question, when studying the effects of visuospatial selective attention on the cortical processing of dynamic (quasi-)rhythmic visual stimulation. Again, we emphasize that both common data analysis approaches taken here can be equally valid and legitimate, yet they likely index distinct neural phenomena. These can occur simultaneously, as in our case, and may represent distinct cortical processes that work in concert to facilitate the processing of visual stimulation at attended locations.
